# Factorial Investigation of Cobalt Retention by Ti and Fe Oxides-Modified Carbon Nanotubes: Multivariate Against Univariate Analysis

**DOI:** 10.3389/fchem.2021.690420

**Published:** 2021-06-16

**Authors:** Ismail Fasfous, Amjad El-Sheikh, Anas Awwad, Yahya Al-Degs, Ebaa Fayyoumi, Jamal Dawoud

**Affiliations:** ^1^ Department of Chemistry, Faculty of Science, The Hashemite University, Zarqa, Jordan; ^2^ Department of Computer Science, Faculty of Prince Al-Hussein Bin Abdullah II for Information Technology, The Hashemite University, Zarqa, Jordan

**Keywords:** multivariate calibration, carbon nanotubes, adsorption, iron oxide, titanium oxide, cobalt

## Abstract

Fe/Ti-oxides-modified-carbon nanotubes CNTs nanocomposites were prepared and tested toward Co removal from solution under different operational conditions. The final performance of the nanocomposites for Co was highly dependent on the type and loaded amount of the oxides. The nanocomposites were characterized by standard methods and the results evidenced that the presence of CNTs hampers the growth of Fe_3_O_4_ and TiO_2_ particles and forming smaller nano-particles leading to better Co removal from solution. Analysis of isotherms at different temperatures indicated that Co retention was two-fold increased upon adding Ti-oxides up to 90.2%. All isotherms were fairly presented using Langmuir-Freundlich isotherm and most surfaces have high heterogeneity particularly after deposition of oxides. The combined influence of the factors was investigated by running a multivariate analysis. An empirical equation was generated by principal component analysis (PCA) for predicting Co retention assuming different relationships and the binary-interaction behavior between factors was the most dominant: Co retention (mg/g) = 5.12 + 1.25Conc + 1.47Temp − 1.38CNT% − 6.03Ti% − 5.03Fe% − 0.01Conc^2^ + 0.12Temp^2^ − 0.55CNT%^2^ − 1.53Ti%^2^ − 3.44Fe%^2^ + 0.17Conc  ×  Temp + 0.07Conc × CNT% + 0.07Conc × Ti% + 0.10Conc × Fe% + 0.21Temp × CNT% + 0.10Temp × Ti% + 0.17Temp × Fe% − 1.67CNT% × Ti% − 1.45CNT% × Fe% − 4.11Ti% × Fe%. The most dominant factors on Co retention were temperature and concentration (positive linear correlation) and the positive interaction between temperature/concentration and temperature/CNTs mass. PCA indicated that the coefficient Temp × CNTs (+0.21) was higher than Temp × Ti% (+0.10). The negative coefficients of Ti/Fe with CNTs (1.45–4.11) indicated better Co retention at higher Ti/Fe loads and lower mass of CNTs. The results support that fact that incorporation of CNTs with Ti/Fe oxides may have a positive synergic impact on Co retention.

## Introduction

Pollution of water resources with heavy metals is of high environmental concern due to their acute and chronic toxicity (Ikehata et al., 2015). Cobalt is an essential micronutrient, constituent of vitamin B_12_, and is also needed for the production of red blood cells (Steven et al., 2000; Simonsen et al., 2012). However, excess intake of cobalt can lead to unwanted health consequences (Simonsen et al., 2012). Therefore, it is necessary to remove Co(II) from ground and surface waters for public health and a clean environment.

Due to their unique properties and applications, carbon nanotubes CNTs and metal oxides modified forms have gained considerable attention (Iijima, 1991; Gao et al., 2009; Gupta and Saleh, 2011; Al-Degs et al., 2012; Li et al., 2013; Mallakpour and Khadem 2016). Much attention is given to CNTs coupled with magnetic iron oxide (i.e., Fe_3_O_4_) nanoparticles to tackle environmental problems due to direct separation and recyclability (Mallakpour and Khadem, 2016), easiness of preparation, and minimal environmental impact and specific affinity (Sadegh et al., 2017). Hu et al. (Hu et al., 2010) have prepared ß-cyclodextrin-modified-CNTs and Fe-oxides-CNTs and the surfaces were tested toward Pb (II) and 1-naphthol. Better retention for pollutants was reported upon adding cyclodextrin to Fe-oxides/CNTs, which attributed to the favorable interaction between hydroxyl groups and hydrophobic cavity of ß-cyclodextrin with Pb (II) and 1-naphthol. In the same line, Hamza et al. (Asmaly et al., 2015) have loaded Fe_2_O_3_ on CNTs fibers following simple impregnation methodology and the final surface was tested toward phenol. The better uptake upon loading Fe-oxides was attributed to nanoparticles of the oxide.

There are two strategies for handling multifactor processes like Co retention, univariate and multivariate analyses (Brereton, 2004). Univariate analysis is often adopted where retention of the solute is monitored as a function of a certain factor (pH for example) while controlling other factors. Following univariate analysis, adsorption isotherms (concentration variations) and thermodynamics (temperature variations) were well reported for many systems. However, multivariate analysis is less tedious as the influence of all factors on the target response is manipulated in little but organized experimental design. Compared with univariate analysis, the interaction between factors and nonlinear behavior of factors on solute retention (response) would be uncovered by the application of multivariate analysis (Brereton,2004; Al-Degs et al., 2012).

The literature encompasses many studies on CNTs/TiO_2_ composites focused on characterization and other chemical properties (Gao et al., 2009; Zhao et al., 2013) and only a few studies on metals uptake. Although CNTs and Fe-oxides-CNTs have been studied for many metals retention, an in-depth study of Co(II) retention by CNTs and oxides-modified-CNTs is still not available. Herein, synthesis of different nanocomposites of CNTs and Fe_3_O_4_ or TiO_2_ and testing their affinity for Co is the principal goal of this research work. Emphasis will be placed on the potential influence of deposited oxides on Co retention. Co adsorption by modified adsorbents and at different operational conditions will be investigated to pick up the best composite for further testing. The combined influence of some significant factors, including Co content, temperature, and amount of loaded Fe and Ti oxides on Co retention from solution, will be modeled using multivariate analysis to uncover the interaction between factors.

## Materials and Methods

### Materials

Carbon nanotubes were purchased from Shenzhen Nanotech port Co. Ltd, Shenzhen, China. They are in the form of multiwalled carbon nanotubes CNTs with dimensions of 5–15 μm length and 10–30 nm external diameter. Cobalt (II) chloride hexahydrate (GCC, United Kingdom), Iron (II) chloride tetrahydrate (BDH, England), Iron (III) chloride hexahydrate (Scharlau, Spain), Titanium (IV) isopropoxide (Sigma Aldrich, United States) were used. All chemicals used were analytical grade unless stated otherwise. Triply distilled water was used to prepare all solutions.

### Synthesis of Fe/Ti-Oxides-CNTs Nanocomposites

In brief, as-received CNTs were refluxed in a 12.0 M HNO_3_ solution for 6.0 h. After cooling, the oxidized CNTs were recovered by filtration, rinsed with enough distilled water to have a neutral filtrate pH, and then dried overnight at 110°C. the acid-treated substrate was abbreviated as O-CNTs. Chemical co-precipitation (Lee et al., 2010) and sol-gel (Gao, Chen et al., 2009) methods were used to prepare CNTs/Fe_3_O_4_ and CNTs/TiO_2_ nanocomposites, respectively. In all preparations, O-CNTs was used due to its better reactivity. For Fe-CNTs*,* 0.90 g of FeCl_3_.6H_2_O/FeCl_2_.4H_2_O (1:3.5, *w/w*) mixture was added to 0.50 g of O-CNTs in 800 ml distilled water with sonication for 2.0 h. The suspension solution was transferred into a three-neck round-bottom flask and stirred under the purge of nitrogen gas. 10 ml of 7.0 M NH_4_OH solution was added to the suspension and kept stirring for 30 min to yield a black colloidal solution. The colloidal solution was then filtered through 0.45 μm micropore membranes, washed with water several times, dried at 100°C for 2.0 h, and labeled as Fe-CNTs (31.8% Fe-oxide). The same procedure was repeated to prepare Fe-CNTs (48.2% Fe-oxide) and Fe-CNTs (18.9% Fe-oxide) composites. All samples were then calcined at 440°C for 4.0 h to get crystalline iron oxide in the nanocomposites as confirmed by XRD. All samples were ground into a powder and stored in a desiccator until used. A control sample was prepared as outlined earlier but in the absence of the substrate and labeled as Fe_3_O_4_. For Ti-CNTs nanocomposites*,* 0.10 g of O-CNTs was dispersed into a 30 ml of isopropanol/water (1:10, *v/v*) solution under sonication for 2.0 h. A 21.4 ml of titanium isopropoxide/isopropanol (1:5.27,*v/v*) was added dropwise into the suspension under stirring. After 2 h, the solid precipitate was then filtered, washed with ethanol/water (2:1,*v/v*) solution several times, dried at 100°C for 1.5 h, and labeled as Ti-CNTs (90.2% Ti-oxides). The same procedure was repeated using different levels of Ti concentration to prepare the following composites Ti-CNTs (87.4% Ti-oxides), Ti-TiO_2_ (82.1% Ti-oxides), Ti-CNTs (69.6% Ti-oxides), respectively. The samples were then calcined, ground, and stored as described earlier. A control sample was also prepared but in the absence of O-CNTs and labeled as TiO_2_. In summary, eleven solid adsorbents were prepared to test Co removal from solution under different operational conditions.

The mass ratio Fe-CNTs was calculated based on the measured mass of O-CNTs and the theoretical yield of Fe_3_O_4_ upon mixing FeCl_3_.6H_2_O with FeCl_2_.4H_2_O in the basic medium (ammonium hydroxide) as shown in chemical equation. The stoichiometric ratio is 2:1 (Fe^3+^/Fe^2+^).

$$2Fe^{3+}\;+\;Fe^{2+}\;+8OH^{-}\to \;Fe_{3}O_{4}\;+\;4H_{2}O$$ (1)

While the mass ratio Ti-CNTs was calculated based on the theoretical yield of TiO_2_ upon reaction titanium isopropoxide with water to deposit TiO_2_ as shown in reaction below

$$Ti{\left(OCH{\left(CH_{3}\right)}_{2}\right)}_{4}\;+\;2\;H_{2}O\;\to \;TiO_{2}\;+\;4{\left(CH_{3}\right)}_{2}CHOH$$ (2)

### Characterization of Nanocomposites and Determination of Cobalt

Specific surface area (SSA) and surface functional groups were measured following standard procedures as described elsewhere (El-Sheikh, 2008). Fourier transform infrared spectra (FTIR) of samples in a range of 4,000–400 cm^−1^ were recorded using a Bruker vertex-70 spectrometer (Ettlingen, Germany). The quantitative determination of Co was performed by using flame atomic absorption spectrometer (iCE 3000 series, Thermo Scientific, United States) under the following instrumental conditions: λ = 240.7 nm, Lamp current = 75 mA, Slit width = 0.2 nm, Burner height = 7 mm, Acetylene flow = 1 L min^−1^, Airflow = 10 L min^−1^. Identification of deposited oxides was carried out using powder X-ray diffraction [X-ray diffractometer (XRD-6000), Shimadzu, Japan] XRD-6000. BARNSTEAD/Thermolyne furnace (Barnstead International, United States) operated up to 1,200°C was used to anneal the composites under inert atmosphere.

### Adsorption Isotherms

In the current study, effects of concentration, temperature, and amount of loaded oxides on Co retention were investigated while keeping pH, mass of adsorbent, and contact time at specified levels. Concentration-variation isotherms were carried out as described elsewhere (Al-Degs et al., 2012). Briefly, A 5.0 mg (±0.0001 g) of dried nano-adsorbent was contacted with 10.0 ml solution containing various concentrations of cobalt (1, 2, 3, 4, 5, 7.5, 10, 15, 20, 25, 30, 35 and 40 mg/L) in 30 ml screw caps vials to prevent solvent vaporization. The vials were agitated in a water bath shaker for 24.0 h. The equilibrium time (24.0 h) was measured from separate kinetic runs, which was found reasonable for all adsorbents. After completion of thermostat shaking (±1°C), mixtures were left to settle for another 1 h at the selected temperature to get a clean suspension solution. Then, a 3.0 ml of clean supernatant was withdrawn for Co quantification bythe flame atomic absorption spectrometer. Co removal by all adsorbents was carried out at pH 6.4 (±0.2) to avoid serious precipitation with OH^−^ ions and prevent possible leaching of Fe or Ti ions from the modified surface in the solution which would limit the practical application the surfaces (Oliveira et al., 2002). Adsorption isotherm was measured at three temperatures (30.0, 40.0, and 50.0°C) to study the thermodynamic of the process. To ensure the reliability of the data, blank samples were prepared (without adding nano-adsorbent) and handled in parallel for each test. The pH experiments were performed in the same procedure described above at a cobalt concentration of 20 mg/L and temperature of 30 ± 1°C. In summary, 33 isotherms were measured to study the effect of concentration, temperature, and loaded oxides on Co removal.

### Modeling Co Retention from Solution: Isothermal and Thermodynamic Behaviors

The amount of Co adsorbed *q*_*e*_ (mg/g) was estimated from equilibrium concentration in solution *C*_e_ (mg/L) as following:

$$q_{e}=\frac{\left(C_{o}-C_{e}\right)V}{m}$$ (3)

Where *C*_*o*_, *V*, and *m* stand for initial concentration of *C*_*o*_ (mg/L), volume of solution (ml), and *m* is the mass of adsorbent (g). For more validation of the proposed procedure, certain isotherms were repeated and a relative error of 2–5% was reported indicating the acceptable repeatability of the procedure. Five isotherms were used to represent equilibrium data points including Langmuir (Allen et al., 2004), Freundlich (Freundlich, 1906), Langmuir-Freundlich (Jeppu and Clement, 2012), Temkin (Temkin and Pyzhev, 1940), and Dubinin-Radushkevich (El-Sheikh et al., 2011). The parameters of the models were estimated following nonlinear regression analysis using Origin lab 8^®^ computer program. Modeling of isotherms and estimation of thermodynamic parameters are outlined in Supplementary Material.

### Multivariate Analysis of Co Retention From Solution: Combined Influence of Factors

Unlike univariate analysis (Section *Modeling Co Retention From Solution: Isothermal and Thermodynamic Behaviors*), multivariate analysis can handle large size of adsorption data to uncover the interaction between factors. Adsorption of Co was tested using 11 adsorbents while changing five factors with a total of 429 tests.

As a multifactor process, Co retention by all tested adsorbents and at different factors would be presented by the empirical equation:

$$\begin{array}{l} Co\;retention\;\left(mg/g\right)=\;b_{0}+\;b_{1}Conc\;+b_{2}Temp+b_{3}CNT\%\;\\ +b_{4}Ti\%+b_{5}Fe\%+b_{11}Conc^{2}+b_{22}Temp^{2}+b_{33}CNT{\%}^{2}+b_{44}Ti{\%}^{2}+b_{55}Fe{\%}^{2}+b_{12}Conc\\ \times Temp+b_{13}Conc\times CNT\%+b_{14}Conc\times Ti\%+b_{15}Conc\times Fe\%+b_{23}Temp\times CNT\%+b_{24}Temp\\ \times Ti\%+b_{25}Temp\times Fe\%+b_{34}CNT\%\times Ti\%+b_{35}CNT\%\times Fe\%+b_{45}Ti\%\times Fe\% \end{array}$$ (4)

Where *b*_*0*_, (*b*_*1*_ *–b*_*5*_), (*b*_*11*_ *–b*_*55*_), and (*b*_*12*_–*b*_*45*_) represented the intercept, coefficients of linear terms, coefficients of quadratic (non-linear) terms, and interaction terms, respectively. As indicated in Eq. 4, modeling Co retention while changing other factors is a complex process as linear, quadratic, and interaction terms are included to handle the process. Linear terms assign the factor(s) that are linearly dependent on Co retention regardless of the variations in the levels of other factors. The quadratic terms are also necessary to account for nonlinear behavior that may involve in the process, for example, retention of Co by Ti-CNTs may not be linearly correlated with loaded amounts (i.e, non-linear correlation). Interaction terms are also necessary to account for any synergetic influence among factors, for example, better Co removal would achieve when the surface is highly loaded with Ti-oxides but at high temperature. The experimental design was placed in matrix **C** while the dependent factor (Co retention values) was placed in vector *q* to build the following equation (Brereton, 2004):

q=Cb (5)

The dimensions of *q*, *C*, and *b* were 429 × 1, 429 × 22, and 22 × 1, respectively (429 tests and 22 coefficients related to the significance of factors on the process). Using PCA, matrix C was decomposed into smaller matrices which were then used to find *b* and build the model the complex of the relationship between *C* and *q* and finally to find the coefficients of Eq. 4 (Brereton, 2004). Initially, Eq. 4 was solved by including linear terms only (*b*_*1*_ *-b*_*5*_ besides intercept *b*_*0*_). In the next step, the model was created by adding quadratic terms and finally by adding interaction terms. The best model was the one that accurately predicts Co-retention from the experimental design. PCA was carried out using home-made codes based on the algorithm outlined in the literature (Brereton, 2004). The quality of fit for models was estimated by finding relative error of prediction (Brereton, 2004):

$$REP\%=100\times {\left(\frac{\sum_{i=1}^{m}{\left(q_{i,pred}-q_{i,measur}\right)}^{2}}{\sum_{i=1}^{m}{\left(q_{i,measur}\right)}^{2}}\right)}^{1/2}$$ (6)

Where *q*_*measur*_, *q*_*prd*_, and *m*, are the measured Co retention value (mg/g), the predicted Co retention value (mg/g) by PCA, and the number of tests, respectively. Before running PCA, the levels of factors were properly coded to get a uniform significance of all factors on Co retention and to end up with an accurate estimation of the coefficients (Brereton, 2004). Retention values were (429 data) were also mean-centered prior to PCA.

## Results and Discussion

### Physicochemical Properties of CNTs and Modified Forms

For a better assessment of the surfaces, detailed characterization tests were carried out which was necessary to evaluate the nature of interaction with cobalt in solution. Table 1 summarizes the main physicochemical parameters along with Co retention for each adsorbent.

**TABLE 1:** Physicochemical parameters of tested absorbents along with maximum affinities toward Co retention from solution.

Nanocomposites^a^	CNTs%^b^	Fe%^b^	Ti%^b^	SSA^c^ m^2^/g	Saturation value (mg/g)^d^	Heterogeneity index^e^	Functionality^e^ (mmol/g)		
							a	b	c
CNTs	100	—	—	193.19	13.86	1.13	4.70	3.92	1.96
O-CNTs	100	—	—	207.28	15.27	0.75	1.88	9.80	4.90
TiO_2_	—	—	100%	122.12	10.42	0.66			
Ti-CNTs (69.6%)	30.4	—	69.6	189.13	16.56	2.14	3.76	5.88	3.92
Ti-CNTs (82.1%)	17.9	—	82.1	179.31	21.43	1.02	2.82	4.90	1.96
Ti-CNTs (87.4%)	12.6	—	87.4	171.16	24.63	1.18	2.82	3.92	1.96
Ti-CNTs (90.2%)	9.8	—	90.2	168.61	29.80	1.71	1.88	2.94	0.98
Fe_3_O_4_	—	100	—	129.10	7.68	0.88			
Fe-CNTs (18.9%)	81.1	18.9	—	185.19	13.30	1.10	3.76	6.86	3.92
Fe-CNTs (31.8%)	68.2	31.8	—	179.31	21.16	1.88	3.76	5.88	2.94
Fe-CNTs (48.2%)	51.8	48.2	—	176.51	20.32	1.62	2.82	4.90	1.96

a Values between brackets indicated the ratios the nano-materials in the final mix.

b Percentages were calculated based on limiting reactant of the reagents involved in synthesis of Fe_3_O_4_ and TiO_2_.

^c^SSA is determined using methylene adsorption method.

d Both saturation values and heterogeneity indices were estimated from Langmuir-Freundlich isotherm equation measured at 50°C, 5 mg adsorbent, Co range 1–40 mg/L, pH 6.4, and shaking time 24 h.

e Based on Bohem’s method, a total basic group (mainly ketonics and amines), b total acidic groups (mainly alcoholic, phenolic and carboxylic acid groups) and c carboxylic acid group.

Upon oxidation, the contents of acidic groups were increased, and the maximum increase was seen in phenolic content. For O-CNTs, the role of acidic groups for Co retention was not significant as only 5% of the carboxylic groups would involve in the process assuming 1:1 complexation with Co^2+^ ions. In fact adding Fe and Ti to CNTs has ended up with selective surfaces able to attract more Co ions. The involvement of functional groups for attracting the particles Ti and Fe oxides was highly possible as supported by the lower contents of functional groups with loaded oxides (Chiang et al., 2011; Martínez et al., 2011). For Fe-CNTs, the load of Fe% was increased up to 48.2% leading to a reduction of 50% in original acidity while the content of basic groups was increased from 1.88 to 2.82 mmol/g. In fact, the large reduction in acidity upon Fe deposition may support the selective interaction between ionisable carboxylic groups with the particles of Fe_3_O_4_. The same behavior seems to be repeated with TiO_2_ particles where a mix of 90.2% Ti resulting in a 70% reduction in the acidity while total basicity was unchanged.

All surfaces exhibited a lower surface area against O-CNTs due to the accumulation of oxides and blocking the micropores, filling up the gaps between CNTs and clogging nanotube openings, thereby reducing the nanocomposite surface area as theorized elsewhere (Gao et al., 2009; Lio et al., 2011). Interestingly, pure Fe and Ti oxides have a modest affinity which supported the fact that spreading both oxides particles (especially TiO_2_) over CNTs has improved Co. Heterogeneity index (Table 1), indicated a better homogeneity of modified surfaces. Ti-oxide has generated better surfaces for Co with maximum retention of 29.80 mg/g at loadings of 90.2%. On the other hand, more Fe-oxide was loaded (48.2%) to reach Co retention of 20.32 mg/g.

The adsorption capacity of Ti-CNTs (90.2%) was notably higher than other adsorbents reported in the literature. Retention values of 8.84, 8.78, 16.26, 72.43 mg/g were reported for iron oxide-MWCNTs (Wang et al., 2011), polyacrylic-MWCNTs (Chen et al., 2012), hydroxyapatite-MWCNTs (Liu et al., 2013), and β-cyclodextrin-graphene oxide (Song et al., 2013), respectively.

### IR Measurement and XRD Pattern of the Active Composites

**FIGURE 1:**
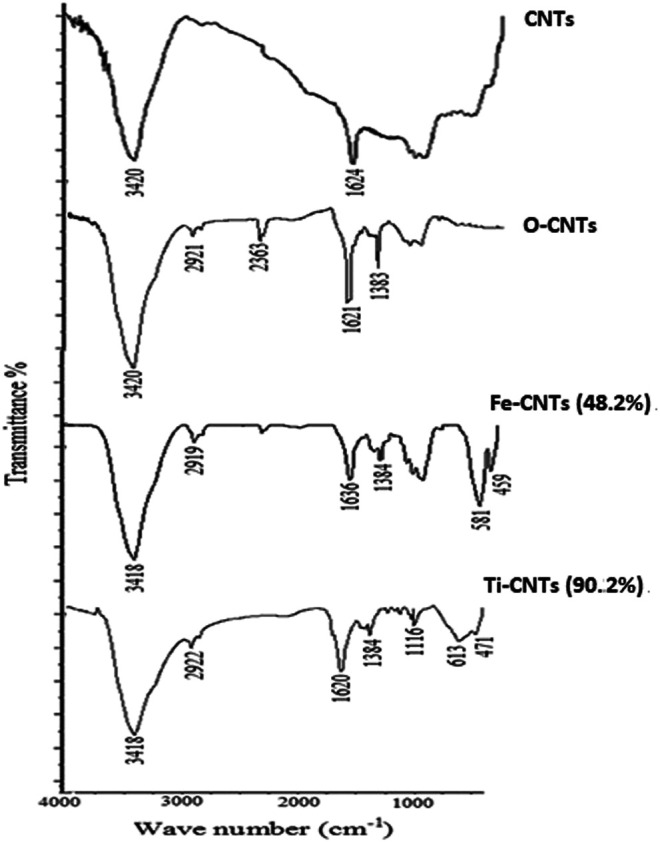
IR spectra of oxides-loaded-CNTs beside original substrates.

IR analysis was made to most effective surfaces as shown in Figure 1.

Two IR bands appeared in CNTs which positioned at 3,420 and 1,624 cm^−1^ and mainly attributed to aromatic phenolic and carboxylic surface groups. Upon oxidation, the earlier bands were increased indicating a more acidic surface. The short band that appeared at 2,921 cm^−1^ was attributed to vibrations of -OH in a carboxylic acid. In general, the spectra of oxides-modified-CNTs exhibited a decrease in the intensity of absorption peaks over 1,630–1,780 cm^−1^ and 3,200–3,550 cm^−1^ against the original substrate which obviously reflected the special linkage between particles of oxides with carboxylic/phenolic surface groups. The IR spectra of Ti-CNTs showed a low intensity of 1,113 cm^−1^, which was assigned to the Ti-O-C bond (Wang et al., 2011). For Fe-CNTs, the sharp two peaks that appeared at 581 and 459 cm^−1^were evidenced by the vibrations of the Fe-O bond [26]. On other hand, the intensity of absorption peaks at 400–700 cm^−1^were increased upon adding more oxides.

**FIGURE 2:**
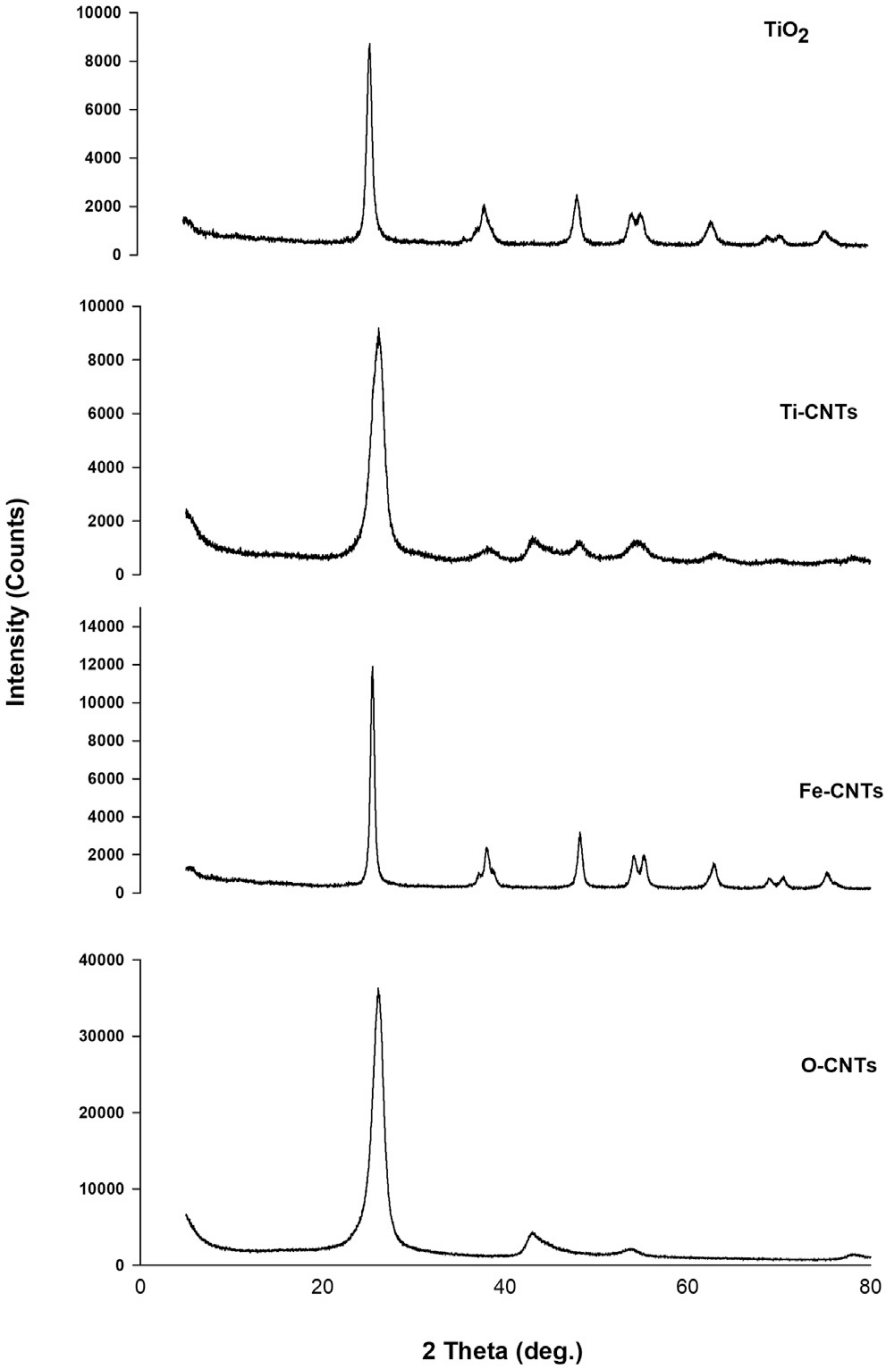
XRD of some main oxides-modified-CNTs surfaces and O-CNTs.

Identification of both oxides was further studied by XRD. Figure 2 shows XRD patterns of main samples.

As indicated in Figure 2, the characteristic peaks of O-CNTs were seen at 2θ 26.04 and 42.80° which are assigned to (002) (100) planes of reflection, respectively (Chen et al., 2011). Upon loading Fe-oxides, the following peaks of variable intensities were observed: 26.12, 30.20, 35.64, 42.96, 53.96, 57.20, and 62.84°. The intrinsic XRD peaks of O-CNTs still detected even after loading large amounts of the oxide. The other diffraction peaks are consistent with the JCPD standard XRD patterns for magnetite Fe_3_O_4_ with cubic crystal system (Wang et al., 2011) (89–3,854, 2θ = 30.088, 35.439, 43.07, 53.432, 56.958, 62.546°), and gamma hematite (γ-Fe_2_O_3_) (89–5,892, 2θ = 30.266, 35.651, 43.332, 53.766, 57.319, 62.949°). However, the XRD pattern of iron oxide (not provided) alone cannot distinguish between hematite and magnetite phases where both have practically indistinguishable patterns (Sun et al., 2005; Wang et al., 2011). It should realize that the peak intensities of O-CNTs decrease after combining with Fe_3_O_4_ nanoparticles. For TiO_2_, many sharp peaks were centered at: 25.46, 37.88, 48.16, 53.96, 55.06, 62.8, 68.8, 70.34, and 75.08°. The strong peaks were seen at 25.46 and 48.16° supporting the formation of anatasephase (Lu et al., 2013; Zhang et al., 2015). The XRD pattern of CNTs loaded with TiO_2_ indicating the following peaks at 2θ: 26.14, 37.62, 42.66, 48.00, 53.92, 62.58, and 69.86°. As was the case in CNTs-Fe, the characteristic peaks of the substrate are still seen, however, the main anatase peak (25.46°) may overlap with the intense peak of O-CNTs (26.04o). The average crystallite size was estimated from the corresponding intensive diffraction peak using Scherrer’s equation (Scherrer, 1918): $d=\frac{K.\lambda }{\beta \cos\theta }$, where ß is the full width at half maximum intensity in radians,θ is the diffraction angle of the XRD peak in degrees, and *K* is the Scherrer’s constant (0.91) (Liu et al., 2013). The results indicated that all adsorbents were in nano-size with diameter of 5.64, 6.33, and 7.32 nm for O-CNTs, Fe-CNTs, and Ti-CNTs, respectively. As indicated from the diameters, deposition of oxides has increased the diameter of the original substrate, however, it remains in the nano-range (1–100 nm). Due to the intense overlap between the O-CNTs (26.04°) and TiO_2_ (25.46°), the average crystallite size of the Ti-CNTs was then calculated based on the peak at position 48.16°, which has negligible interference the substrate. The results showed that the average crystallite size of the composites was less than that of pure TiO_2_ (average diameter 16.5 nm) indicating that the presence of CNTs in the nanocomposites hinders the particle growth and leads to a smaller TiO_2_ nanocrystallite size (Gao et al., 2009; Li et al., 2011; Wang et al., 2011).

### Adsorption Isotherms: Isothermal and Thermodynamic Behavior

#### pH and Optimum Co Retention

**FIGURE 3:**
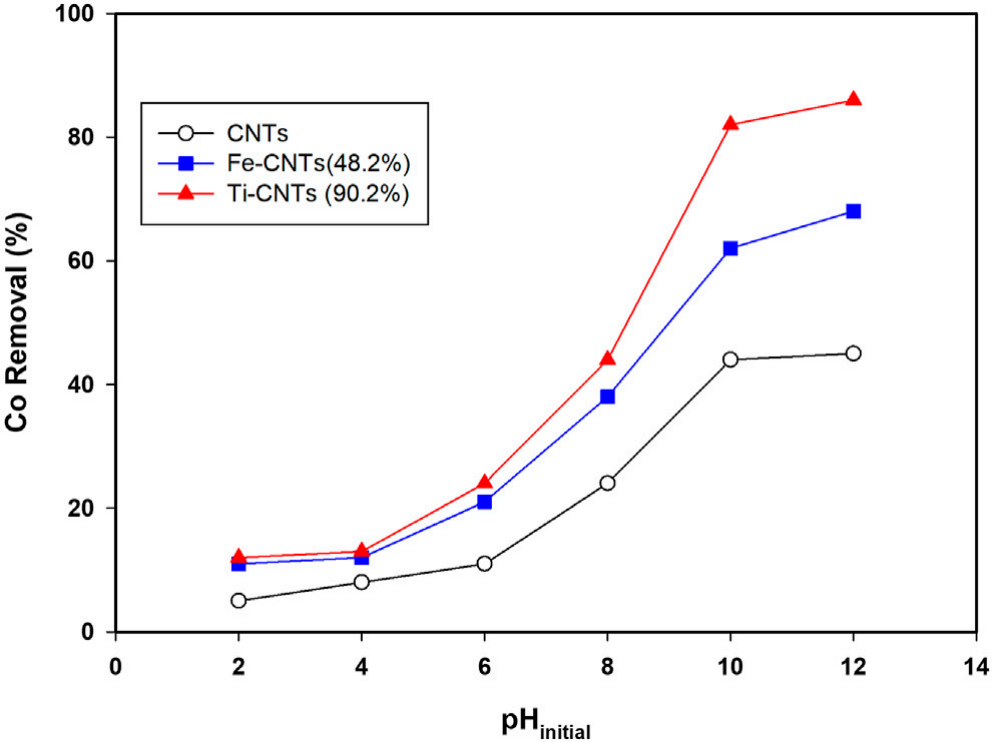
Influence of solution pH on Co removal (Conditions: mass 5.0 mg, Co level 20 mg/L, temp = 30°C, and shaking time 24 h).

Initial studies indicated that pH has a significant influence on Co removal and this was noticed for all surfaces. For better comparison, all isotherms were carried out at certain pH. Figure 3 shows the removal behavior of Co over the pH range (2.0–12.0).

The removal of cobalt (%) increases slowly at pH ≤ 4.0, then quickly at pH 6.0–10.0, sustains a steady or slight increase at pH ≥ 10.0. At low pH (<4.0), both cobalt species and the surface of the nanocomposite are positively charged, which explains the low removal efficiency of the nanocomposite. Over the range of 4.0–9.0, the surface of the nanocomposite becomes more negatively charged depending on the functional groups being ionized, and the electrostatic interaction between cobalt ions and nanocomposite surface increases, leading to more adsorption. At pH > 9, retention is mainly governed by precipitation in the form of cobalt hydroxide as theorized elsewhere (Wang et al., 2011; Hongqin et al., 2012; Li et al., 2013; Chen et al., 2015; Wang et al., 2015).

#### Co Retention and Loadings of Oxides: Univariate Analysis

**FIGURE 4:**
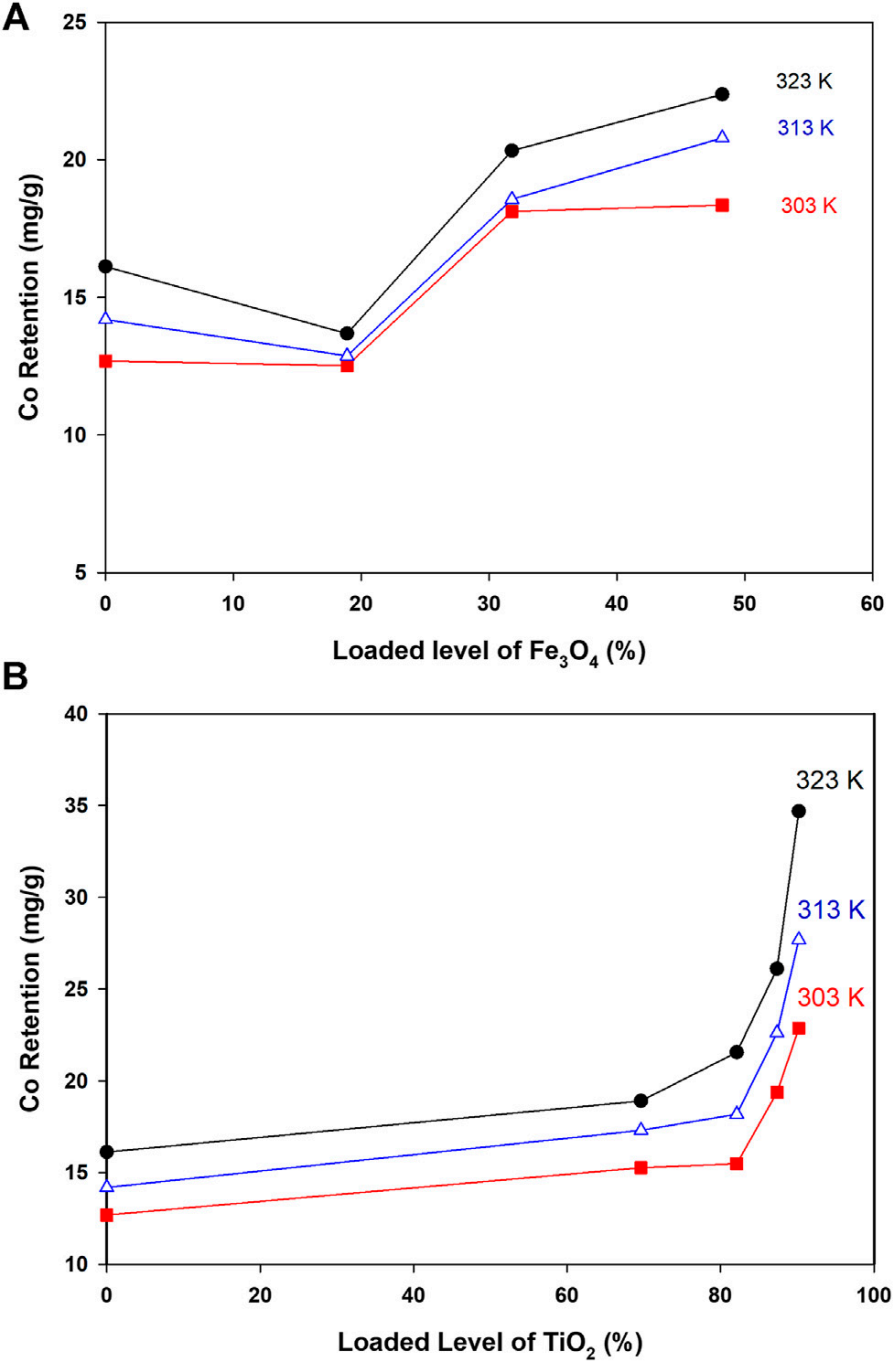
Co retention as a function of loaded oxides and at different temperatures (Conditions: Co level 40 ppm, mass 5.0 mg, pH 6.4, and shaking time 24 h).

As indicated in Figure 4, retention of Co ions from solution was significantly improved upon adding Ti and Fe oxides to CNTs. Retention of Co as a function of loaded oxides was studied at different temperatures, and the main results are depicted in Figure 4.

As indicated in Figure 4A, retention of Co by loaded surfaces is influenced by the loaded amount. In general, to get better Co uptake, the surface should be loaded with amounts higher than 30%. The interesting point in Figure 4A was the reduction in Co uptake by Fe-CNTs (18.9%) and at 50.0°C. For all tested surfaces, uptake of Co improved at higher temperature and best efficiency was observed at surface oxide loading of 48.2%. In fact, the relationship between Co uptake and loaded oxides is not purely linear and this would be attributed to the involvement of other factors like nature of interaction with composites, the extent of pore-filling, and mode of distribution of nanoparticles by the surface. For Ti-CNTs, better linearity between Co retention with loaded amounts was observed. In all cases, Co retention was increased with loaded TiO_2_ and the maximum uptake was 34.7 mg/g at surface loading of 90.2%. The main conclusion that drawn from Figure 4 is that Ti-CNTs outperformed Fe-CNTs for Co uptake and more Fe-oxide was needed to get better Co retention. Comparable behavior was in the literature on the synergistic influence of metal oxides-CNTs toward adsorption from solution (Oliveira et al., 2002; Wang et al., 2007; Hu et al., 2010; Li et al., 2011; Wang et al., 2011). For example, Li et al. (Li et al., 2011) reported that the presence of CNTs in the composites enhanced the textural properties of the CNTs/TiO_2_ composites compared to pure TiO_2_ aggregated particles, and by providing an open structure and high surface area support for the formation of metal oxide films. Table 2 shows a comparison of the maximum cobalt retention of the prepared nanocomposites with other adsorbents in the literature.

**TABLE 2:** Comparison of Co retention of prepared nanocomposites and the previous studies in the literature.

Sorbent	Retention capacity, (mg/g)	Condition				Ref
		pH	Temp K	Ionic strength	m/v g/L	
Iron oxide/CNTs	2.88	6.4	293	0.01 M	0.50	Wang et al. (2011)
CNTs	2.60	9	293	—	5.00	Stafiej and Pyrzynska (2007)
Activated carbon	1.2	6	303	—	180.0	Sulaymon et al. (2009)
Magnetite/graphene oxide	12.98	6.8	303	0.01 M	0.40	Liu et al. (2011)
β–Cyclodextrin/graphene oxide	72.43	6	303	0.01 M	0.10	Song et al. (2013)
Titanate/graphene oxide	81.3	6	293	0.01 M	0.17	Wang et al. (2015)
Fe_3_O_4_/bentonite	18.76	8	293	—	0.10	Hashemian et al. (2014)
O-CNTs	15.27	6.4	323	—	0.50	This work
Fe-CNTs (48.2%)	20.32	6.4	323	—	0.50	This work
Ti-CNTs (90.2%)	29.80	6.4	323	—	0.50	This work

The remarkable affinity of Ti-CNTs over the Fe-CNTs toward cobalt species can be explained by the difference in the surface properties of the metal oxides such as crystal lattice structure, particle size, morphology, the quantity of the oxide, and functional groups (mainly hydroxyl), which probably results in the penetration of different amounts of Co ions from the surface into aggregates of the nanoparticles (Tamura et al., 1997). had reported that the adsorption ability of a series metal oxides for cobalt ions in solution increases in this order Al_2_O_3_ < Fe_2_O_3_ < TiO_2_ < Fe_3_O_4_< MnO_2_. They reported a positive correlation between cobalt adsorption capacities and increasing the electronegativity of the metal cation in the oxide lattice structure, which enhances the acid surface hydroxyl group deportation before bonding with Co^2+^.

#### Isotherms and Thermodynamic Parameters

**FIGURE 5:**
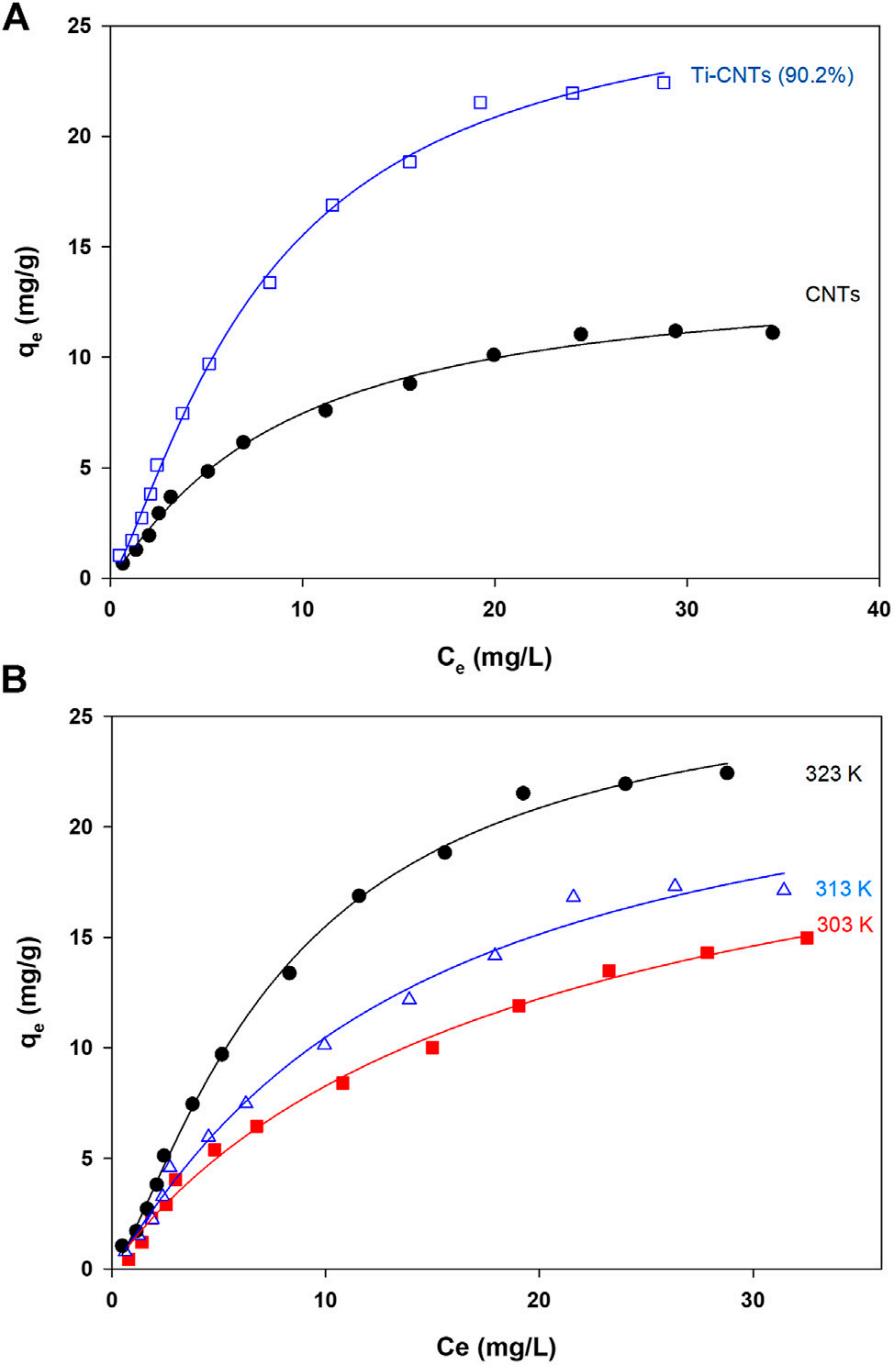
Adsorption isotherms A: Co ions adsorption by CNTs and Ti-CNTs (90.2%) B: Adsorption isotherms of Co by Ti-CNTs (90.2%) at different temperatures (Lines passing through experimental points representing Langmuir-Freundlich modeling).

Measurement of Co isotherms at different temperatures provides crucial information about the physiochemical properties of nanocomposites and instant insights on the sorption process. The experimental data (*C*_*e*_ and *q*_*e*_) were analyzed by different isotherms and at three temperatures to find thermodynamic parameters. The number of measured isotherms was high (33 isotherms), however, the analysis was limited to Ti-CNTs (90.2%) as the best efficiency was reported for this material. For comparison purposes, adsorption isotherms of Co by CNTs were also included. Typical isotherms are depicted in Figure 5 while isotherms and other thermodynamic parameters are presented in Table 3.

**TABLE 3:** Parameters of the tested isotherms and thermodynamic values of Co retention by CNTs and Ti-CNTs.

Model^a^	CNTs	Ti-CNTs (90.2%)
Langmuir	*K*_*L*_ = 0.09	*K*_*L*_ = 0.074
	*q*_*m*_ = 15.3	*q*_*m*_ = 34.7
	*X* ^2^ = 0.3	*X* ^2^ = 1.0
Freundlich	*K*_*F*_ = 1.98	*K*_*F*_ = 3.39
	*n*_*F*_ *=* 0.52	*n*_*F*_ *=* 0.59
	*X* ^2^ = 1.7	*X* ^2^ = 4.0
Langmuir-Freundlich	*K*_*LF*_ = 0.11	*K*_*LF*_ = 0.21
	*q*_m_ = 13.86	*q*_m_ = 29.83
	*n* = 1.13	*n*= 1.72
	*X* ^2^ = 0.2	*X* ^2^ = 0.7
Temkin	*K*_*T*_ = 1.17	*K*_*T*_ = 1.18
	*b* = 1.1 × 10^−3^	*b* = 2.3 × 10^−3^
	*X* ^2^ = 2.2	*X* ^2^ = 3.5
Dubinin–Radushkevich	*q*_*m*_ = 10.2	*q*_*m*_ = 29.9
	B = 2.2	B = 2.6
	E(kJ/mol) = 4.8	E(kJ/mol) = 4.3
	*X* ^2^ = 3.7	*X* ^2^ = 4.9
Thermodynamics^b^	ΔH° = 33.37 kJ/mol	ΔH° = 10.56 kJ/mol
	ΔS° = 174.3 J/k.mol	ΔS° = 102.1 J/k.mol
	ΔG° = −22.96 (kJ/mol at 50°C)	ΔG° = −22.44 (kJ/mol at 50°C)

a The collected parameters were obtained by fitting adoption data (C_e_ and q_e_) by the models following non-linear regression procedures. Conditions: 50°C, 5.0 mg adsorbent, Co range 1–40 mg/L, pH 6.4, and shaking time 24 h.

b Thermodynamic parameters were estimated as outlined earlier.

As shown in Figure 4, the typical L2-shape of isotherms was obtained based on Giles and Smith classification of isotherms (Giles et al., 1974). In L2 isotherm, surface concentration is steeply raised at low Co content in solution and then increased at the higher concentrations (Giles et al., 1974). This may indicate the high affinity between Co ions and the surface. Indicial L2-shapes were reported for Co and other heavy metals retention by natural silicate minerals (Giles et al., 1974). In general, L2-type isotherms are often reported for ionic solutes adsorption with weak-solvent-competition (Giles et al., 1974). The parameters of isotherms and quality of fit *X* ^2^ are provided in Table 3. In general, Langmuir and Langmuir-Freundlich isotherms were workable for presenting retention behavior of Co by CNTs and Ti-CNTs with *X* ^2^ values 0.2–1.0. The maximum retention values (*q*_*m*_) of Co that predicted by the Langmuir model were slightly higher for CNTs and notably higher for Ti-CNTs when compared to those depicted in Figure 5A. However, Langmuir-Freundlich isotherm was reasonably predicted maximum retention values indicating the workability of the model which can account for the presence of homogeneous and heterogeneous surfaces (Jeppu and Clement, 2012).

The magnitudes of *q*_*m*_ also confirmed the enhancement of Co retention upon modification, 13.86 and 29.83 mgCo/g CNTs and Ti-CNTs, respectively. In the same line, both *K*_*L*_ and *K*_*LM*_ reflected the higher affinity of Co to the surface after deposition of Ti-oxides. Although Langmuir and Langmuir-Freundlich isotherms were workable for presenting Co retention from solution, checking the workability of other isotherms is still necessary. As indicated in Table 3, Freundlich isotherm was not effective as observed for the earlier isotherms for presenting Co retention. *X* ^*2*^ values were 1.7 and 4.0 for CNTs and Ti-CNTs, respectively. The limited application of Freundlich isotherm was expected as this model works well for highly heterogeneous surfaces. Although the model was not highly applicable in this case, *K*_*F*_ indicated a favorable Co retention by both surfaces (*K*_*F*_ > 1). Temkin isotherm, in fact, was found acceptable for presenting Co retention by both surfaces as indicated from *X* ^*2*^ values (2.2–3.5). Moreover, the variation in adsorption energy in the model (i.e., *b* parameter) was positive for both systems which indicated that Co retention from solution was an endothermic process. The three-parameter isotherm (Dubinin-Radushkevich) was also workable for presenting Co retention by both surfaces with acceptable *X* ^*2*^ values, however, *q*_*m*_ values were not in agreement with experimental ones. For example, the predicted *q*_*m*_ of Co retention by CNTs was 10.2 mg/g and this value is lower than the experimental value 13.0 mg/g (Figure 5). In fact, Langmuir-Freundlich isotherm was more accurate in predicting *q*_*m*_ Co retention in both systems. The energy parameter of Dubinin-Radushkevich isotherm (B) also confirmed the better affinity of Co toward Ti-CNTs. In fact, binding energies of Co with surfaces were 4.8 and 4.3 kJ/mol for CNTs and Ti-CNTs, respectively. Thermodynamic parameters provide valuable information about the spontaneity, randomness and heat change in the sorption process (Liu, 2009; Anastopoulos and Kyzas, 2016). By plotting a graph of ln*K* vs. (1/*T*) (*K* may be taken as Langmuir or Langmuir-Freundlich constants), thermodynamic parameters were estimated and presented in Table 3. The positive enthalpy change (10.56–33.37 kJ/mol) suggests that Co retention was endothermic, and this was reported for all surfaces. The values of ΔG^°^ are negative (about −23 kJ/mol), which reveal a spontaneous and thermodynamically favorable process. The positive ΔS (102.1–174.3 J/k.mol) revealed high randomness at the solid-liquid interface. Similar results were also reported for Co removal by other adsorbents (Wang et al., 2011; Hongqin et al., 2012; Song et al., 2013; Wang et al., 2015). Thermodynamic and isotherms parameters would be used to elucidate the nature of the interaction of Co with nanocomposite. The heat of Co adsorption is taken as the sum of three energies: the energy needed to break the intermolecular forces between Co and solvent molecules, the energy needed to break the intermolecular forces between Co ions, and the released energy due to bond formation between Co ions and the composite. Accordingly, the following equation would be provided: ΔH_process_ = ΔH_Co-H2O_ + ΔH_Co-Co_ + ΔH_Co-composite_. The first two terms are positives (endothermic processes) while the last one is negative (exothermic process). From Table 3, ΔH_process_ and ΔH_Co-composite_ were (33.37 and 4.8 kJ/mol) for Co retention by CNTs and (10.56 and 4.3 kJ/mol) for Co retention by Ti-CNTs. Accordingly, the sums of ΔH_Co-H2O_+ ΔH_Co-Co_ were 28.57 and for 6.26 kJ/mol, respectively. Simply, 85 and 60% of total energy were used to break down the forces between Co-H_2_O and Co-Co interactions. In fact, interactions between Co ions are not high compared with Co-H_2_O due to the low content of the ion. Both Harkin-Jura and Halsey isotherms were also found of limited application for presenting Co retention by both surfaces and this was excluded the formation of multilayer of Co by the surface.

### Multivariate Analysis of Co Retention

The combined influence of all factors on Co retention will be analyzed by PCA to uncover the interaction between factors or to exclude unnecessary factors from the process. The combined influence of factors on Co removal would be anticipated from the final outputs of PCA analysis. Co retention by different adsorbents and conditions is presented using Eq. 7:

$$\begin{array}{l} \text{Co retention}\left(\text{mg}/g\right)=5.12+1.25\text{Conc}+1.47\text{Temp}-1.38\text{CNT}\%-6.03\text{Ti}\%-5.03\text{Fe}\%\\ -0.01{\text{Conc}}^{2}+0.12{\text{Temp}}^{2}-0.55\text{CNT}{\%}^{2}-1.53\text{Ti}{\%}^{2}-3.44\text{Fe}{\%}^{2}+0.17\text{Conc}\times \text{Temp}+0.07\text{Conc}\times \\ \text{CNT}\%+0.07\text{Conc}\times \text{Ti}\%+0.10\text{Conc}\times \text{Fe}\%+0.21\text{Temp}\times \text{CNT}\%\\ +0.10\text{Temp}\times \text{Ti}\%+0.17\text{Temp}\times \text{Fe}\%-1.67\text{CNT}\%\times \text{Ti}\%-1.45\text{CNT}\%\times \text{Fe}\%-4.11\text{Ti}\%\times \text{Fe}\% \end{array}$$ (7)

The value of intercept (*b*_*o*_) gives an average value of all terms, linear, quadratic, and interaction terms. The coefficients of linear terms (1.25, 1.47, −1.38, −6.03, −5.03) allow for a direct relationship between uptake and a given factor. Quadratic terms (−0.01, 0.12, −0.55, −1.53, −3.44) allow to balance out linear terms and this is necessary for certain systems when the optimum uptake is achieved at a specific level (i.e, nonlinear behavior). Interaction terms (0.17, 0.07, 0.07, 0.10, 0.21, 0.10, 0.17, −1.67, −1.45, −4.11) allow to assess the influence of two factors on uptake and this influence is rarely independent. Value of coefficient, in fact, can tell how significant the factor (or combination of factors) is. For linear terms, the high coefficient of a certain factor indicated its linear influence on Co uptake. If the interaction coefficient of two factors is high, then a higher response would be achieved when these factors are maintained at their higher limits and vice versa. Once the coefficients of Eq. 7 were estimated, then the exact influence of factors on Co retention would be ascertained.

As indicated in Eq. 7, the coefficient of concentration and temperature factors were high and positive (1.25 and 1.47) indicating their positive and linear effect on Co uptake under the tested operational factors. The high and positive values of the earlier factors reflected the high correlation between Co retention and concentration/temperature regardless of the variations in the levels of other factors. Simply, an intense increase in Co retention at higher concentrations is expected whether the temperature is high (323 K) or low (303 K).

For the rest of the factors (CNTs%, Ti%, and Fe%), the high and negative coefficients (−1.38, −6.03, and −5.03) indicating anti-correlation with Co. uptake from solution. Regarding the effect of Ti% and Fe% on Co retention, the high and negative coefficients (−6.03 and −5.03) excluding any positive linearity in the process. In fact, including other terms (quadratic and interaction terms) was necessary at this stage to build the model. Among quadratic terms, the high coefficients of Ti% and Fe% indicated a strong nonlinear correlation between these significant factors and Co retention from solution while changing other factors. The interesting point was the modest linear and quadratic relationship between Co retention and CNT% and this may be attributed to the inner-dependency between CNTs% and amount of loaded oxides.

The interaction among factors has a higher influence on Co retention and this was obvious from the high and negative coefficients of interaction terms, −4.11, −1.67, and −1.45 for Ti% × Fe%, CNT% × Ti%, and CNT% × Fe%, respectively. Moreover, positive coefficients (0.07–0.17) between concentration and other factors were found indicating a high Co retention would be obtained by keeping concentration and other factors at their higher limits. The positive interaction of concentration with other factors was expected due to the strong linear correlation with Co retention. As predicted by PCA, the maximum interaction was between Ti% and Fe% (−4.11) indicating that the best Co retention should be achieved when both factors at their opposite limits (i.e., either at high Ti and low Fe or vice versa). The same behavior is true between CNTs-TI and CNTs-Fe. With high coefficients (0.17–0.21), PCA analysis of the 429 tests predicted favourable Co retention when temperature, concentration, and CNTs maintained at their upper limits. The interaction between CNTs/concentration, Ti/concentration seems to be not promising as indicated by their corresponding coefficients (0.07).

The higher coefficient of Fe/concentration (0.10) compared to Ti/concentration (0.07) would reflect the importance of Fe compared to Ti for better Co retention. In general, the coefficient of Conc × Temp was not high (0.17) which makes the increasing temperature (from 303 to 323) may be unnecessary. The best effect of temperature was noticed at higher CNTs with a positive coefficient of +0.21. Moreover, better Co uptake is expected at high temperature and Fe (+0.17) compared to Ti (+0.10).

**FIGURE 6:**
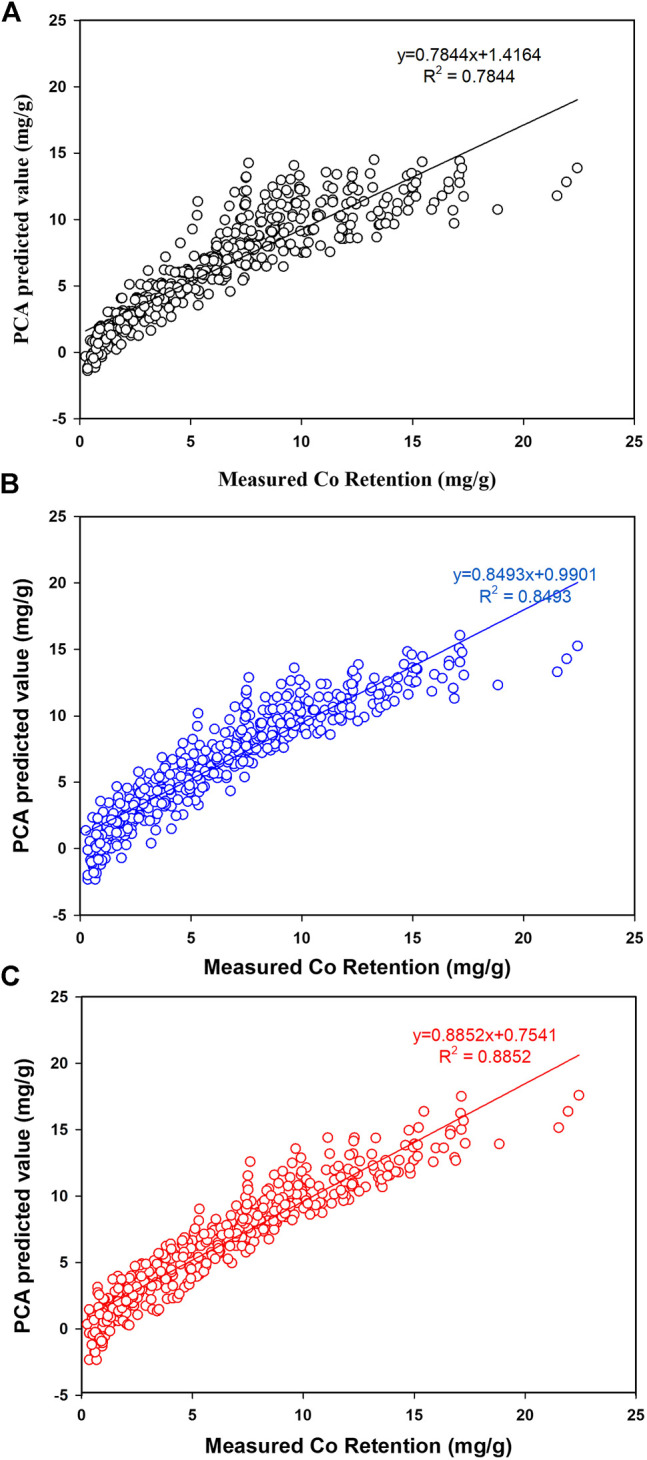
Prediction of Co retention by PCA. A: Linear terms, B: Quadratic terms, C: Interaction terms.

Figure 6 depicts the influence of adding linear, quadratic, and interaction terms on modeling Co retention in all tests and the final REP% are presented in Table 4.

**TABLE 4:** Prediction of Co retention (%REP) by principal component regression while including all possible interactions.

Factors	Linear terms (%)	Quadratic terms (%)	Interaction terms (%)
Conc-Temp-CNT%-Ti%-Fe%	26	22	19

As indicated in Figure 6A, modeling Co retention using linear terms has ended up with a high REP% value of 26% reflecting the complex process and possible interaction between factors. As viewed in Figure 6A, a poor prediction of Co retention particularly at lower retention values (<5.0 mg/g) and negative values were also observed. This observation would be attributed to the significant interaction between factors making linear terms not workable to model the process. A small improvement (Figure 6B) was observed upon including quadratic coefficient (which would help to account for nonlinear correlations) but the overall fitness was modest with *R* ^2^ = 0.8493 and REP% of 22%. Again, the PCA model was not workable for predicting Co retention at lower retentions and this was attributed to the intense interaction between factors. The optimum prediction of Co retention was accomplished when interaction terms were included in the model. Figure 6C confirmed the workability of PCA for simulating Co retention even at low retentions with *R* ^2^ = 0.8852 and REP% of 19%.

Experimentally, the maximum Co retention was 22.4 mg/g and accomplished at the following operational conditions (40.0 ppm, 323 K, CNT 9.8%, and Ti 90.2%). In fact, the high Co affinity at aforementioned conditions agrees with PCA as the levels of Co, Ti, and temperature were at their upper limits and assured the importance of the positive interaction between factors. Although Co retention was relatively high, results indicated that 29% of the ultimate capacity (80 mg/g) was utilized. Accordingly, other experimental factors like pH and ionic strength should be tested for better Co retention from the solution.

## Conclusion

The potential application of many modified nano-adsorbents to remove Co ions from solution was demonstrated. The large size of adsorption tests was analyzed using both univariate and multivariate procedures to model Co retention from the solution. Univariate analysis indicated that Co retention increased with initial concentration, loaded oxides, pH, and temperature. All isotherms were fairly presented using Langmuir-Freundlich isotherm as the model predicted the maximum retention of Co. Thermodynamic studies indicated that Co interaction by all surfaces was a spontaneous and endothermic process. Incorporation of CNTs with TiO_2_ and Fe_3_O_4_ has a synergic impact on Co removal as confirmed by multivariate analysis.

PCA analysis uncovers the interaction among factors that can improve Co retention from solution. The negative coefficients of Ti/Fe with CNTs (1.45–4.11) indicated better Co retention at higher Ti/Fe loads and lower mass of adsorbent. Moreover, the analysis indicated that Ti and Fe should be at their opposite limit to get high Co uptake from solution.

## Data Availability

The original contributions presented in the study are included in the article/Supplementary Material; further inquiries can be directed to the corresponding author.
