# Supplementary material for: Factorial Investigation of Cobalt Retention by Ti and Fe Oxides-Modified Carbon Nanotubes: Multivariate Against Univariate Analysis
Source: Front Chem. 2021 Jun 16;9:690420. doi: 10.3389/fchem.2021.690420 (PMC8241930; doi:10.3389/fchem.2021.690420)
Supplement: Supplementary file 1 [file DataSheet1.docx]

Supplementary Material

# Modeling Co retention by different isotherms and thermodynamic parameters

Langmuir model, which assumed active sites of equal energy, monolayer adsorption, and no solute–solute interactions, is given as ([Allen, McKay et al. 2004](#_ENREF_1)):

$q_{e}=\frac{q_{m}K_{L}C_{e}}{1+K_{L}C_{e}}$ (1)

Where q_m_(mg/g) and K_L_(L/mg) are the maximum retention value of Co and Langmuir parameter or the equilibrium constant ([Allen, McKay et al. 2004](#_ENREF_1)). The empirical Freundlich model, which is based on adsorption by a heterogeneous surface, takes the form ([Allen, McKay et al. 2004](#_ENREF_1)):

$q_{e}=K_{F}C_{e}^{n}$ (2)

Where K_F_(mg/L) is the equilibrium constant indicative of relative adsorption capacity and n is the model exponent which characterizes quasi-Gaussian energetic heterogeneity of the surface. A favorable uptake of Co ions is indicated by higher n values ([Allen, McKay et al. 2004](#_ENREF_1)).The Langmuir-Freundlich isotherm can handle both Langmuir-type and Freundlich-type uptake behavior of Co. This model is a good candidate to model Co removal due to the large variations in the heterogeneity of the nano-size adsorbents stem from deposition of Ti/Fe oxides by CNTs. A common form of the model is given as ([Jeppu and Clement 2012](#_ENREF_6)):

$q_{e}=\frac{q_{m}(K_{LF}C_{e})^{n}}{(K_{LF}C_{e})^{n}+1}$ (3)

Where q_m_ has the same meaning as in Langmuir isotherm and also measure the total number of binding sites available per gram of adsorbent, K_LF_ is the affinity constant for adsorption (L/mg), and n is the index of heterogeneity ([Jeppu and Clement 2012](#_ENREF_6)). Ideally, the value of *n* should be 1.0 for a homogeneous material and much less than unity for heterogeneous surfaces ([Jeppu and Clement 2012](#_ENREF_6)). Based on the energy of adsorption between adsorbent–adsorbate interactions, Temkin presented the following isotherm ([Temkin 1940](#_ENREF_9))

$q_{e}=\frac{RT}{b}\ln(K_{T}C_{e})$ (4)

K_T_ (L/mg) and b (kJ/mol) are the equilibrium binding constant (L/mmol) and the relative heat of adsorption. R and T are the universal gas constant (8.314 J/mol K) and temperature (K), respectively. Porosity and apparent energy of adsorption could be also estimated using Dubinin–Radushkevich isotherm ([El-Sheikh, Al-Degs et al. 2011](#_ENREF_3)):

$q_{e}=q_{m}e^{-B\varepsilon^{2}}$ (5)

The parameter ε is initially estimated from C_e_, ε = [RTln(1+1/C_e_)]^2^.The constant B (kJ^2^ mol^−2^) is related to the free energy of Co adsorption by nano-adsorbents. The apparent energy E (kJ mol^−1^) of adsorption is calculated from B as E=1/(2B)^1/2^ ([El-Sheikh, Al-Degs et al. 2011](#_ENREF_3)). Assessment of models for presenting Co isotherms was carried out by estimating the chi-square value, X^2^ from measured and predicted retentions as following ([Ho 2004](#_ENREF_5)):

$X^{2}=\sum_{i=1}^{N} \frac{(q_{ei,measured}-q_{ei,predicted})^{2}}{(q_{ei,predcited})}$ (6)

Where N is the number of experimental points (13), the model of better performance should have the lowest X^2^. Using the energy parameter of Langmuir isotherm K_L_, the thermodynamic parameters (ΔG^°^, ΔH^°^, ΔS^°^) were calculated ([Liu 2009](#_ENREF_8); [Anastopoulos and Kyzas 2016](#_ENREF_2)):

$\Delta G^{0}=-RT\ln K_{L}$ (7)

And,

$\ln K_{L}=\frac{\Delta S^{0}}{R}-\frac{\Delta H^{0}}{RT}$ (8)

Both R and T were defined earlier. Plotting lnK_L_ against 1/T should end up with straight line and heat of adsorption and entropy of Co removal can be estimated from the slope and the intercept, respectively.

# References

Allen, S. J., G. McKay, et al. (2004). "Adsorption isotherm models for basic dye adsorption by peat in single and binary component systems." Journal of Colloid and Interface Science 280(2): 322-333.

Anastopoulos, I. and G. Z. Kyzas (2016). "Are the thermodynamic parameters correctly estimated in liquid-phase adsorption phenomena?" Journal of Molecular Liquids 218: 174-185.

El-Sheikh, A. H., Y. S. Al-Degs, et al. (2011). "Effect of oxidation and geometrical dimensions of carbon nanotubes on Hg(II) sorption and preconcentration from real waters." Desalination 270(1): 214-220.

Gao, B., G. Z. Chen, et al. (2009). "Carbon nanotubes/titanium dioxide (CNTs/TiO2) nanocomposites prepared by conventional and novel surfactant wrapping sol–gel methods exhibiting enhanced photocatalytic activity." Applied Catalysis B: Environmental 89(3–4): 503-509.

Ho, Y. S. (2004). "Selection of optimum sorption isotherm." Carbon 42: 2.

Jeppu, G. P. and T. P. Clement (2012). "A modified Langmuir-Freundlich isotherm model for simulating pH-dependent adsorption effects." Journal of Contaminant Hydrology 129: 46-53.

Lee, P.-L., Y.-K. Chiu, et al. (2010). "Synthesis of a hybrid material consisting of magnetic iron-oxide nanoparticles and carbon nanotubes as a gas adsorbent." Carbon 48(5): 1397-1404.

Liu, Y. (2009). "Is the Free Energy Change of Adsorption Correctly Calculated?" Journal of Chemical & Engineering Data 54(7): 1981-1985.

Temkin, M. a. P., V. (1940). "Kinetics of Ammonia Synthesis on Promoted Iron Catalysts." Acta Physicochimica URSS 12: 6.
